# Effect of statin therapy on plasma C-type Natriuretic Peptides and Endothelin-1 in males with and without symptomatic coronary artery disease

**DOI:** 10.1038/s41598-020-64795-7

**Published:** 2020-05-13

**Authors:** Timothy C. R. Prickett, Richard W. Troughton, Eric A. Espiner

**Affiliations:** Department of Medicine, University of Otago, Christchurch, New Zealand

**Keywords:** Cardiology, Medical research

## Abstract

C-type Natriuretic Peptide (CNP) and Endothelin-1 (ET-1) have reciprocal roles in maintaining vascular homeostasis and are acutely modulated by statins in human cultured endothelial cells. Whether these actions of statins *in vitro* are reflected in studies *in vivo* is unknown. In a prospective study of 66 subjects with or without post- acute coronary syndrome (ACS), plasma concentrations of bioactive CNP and bio-inactive aminoterminal proCNP (NTproCNP), ET-1, B-type Natriuretic Peptide (BNP) and high sensitivity C Reactive Protein (hsCRP) were measured together with lipids before and at intervals of 1, 2 and 7 days after commencing atorvastatin 40 mg/day - and for a further period of 6months in those with ACS. Plasma lipids fell significantly in all subjects but plasma CNP, NTproCNP and ET-1 were unchanged by atorvastatin. In ACS, baseline hsCRP, BNP and CNP but not NTproCNP or ET-1 were significantly raised compared to values in age-matched controls. The ratio of NTproCNP to CNP was significantly lower in ACS throughout the study and was unaffected by statin therapy. We conclude that conventional doses of atorvastatin do not affect plasma CNP products or ET-1. Elevated CNP after cardiac injury likely results from regulated changes in clearance, not enhanced production.

## Introduction

Statin therapy is widely employed to prevent coronary artery disease and stroke. Statins reduce serum cholesterol and have contributed to reduced morbidity and mortality of cardiovascular disease in the last three decades. Independent of actions on lipids, numerous studies show potentially beneficial (pleiotropic) effects of statins – including anti-inflammatory actions within vascular tissues^1^ – which are important in maintaining endothelial integrity and preventing atherosclerosis^2^. *In vitro* studies suggest that statins may have important beneficial effects on important regulators of vascular structure and function. Among these are the endothelial peptides, C-type Natriuretic Peptide (CNP) and Endothelin-1 (ET-1) both of which are modulated by statins in studies using human vascular endothelial cells in tissue culture^3^.

CNP is a growth factor expressed in the vascular endothelium wherein anti-inflammatory, anti-proliferative and vaso-dilator actions of the mature peptide have vasoprotective roles by reducing intimal injury^4^. While evidence supports a largely paracrine mode of action of CNP, clinical studies show that products of proCNP in plasma (aminoterminal proCNP, NTproCNP, and bio active CNP) are increased in settings of vascular stress in both young adults^5^ and at mid-life^6^ – possibly as an adaptive response to inflammation and/or shear stress^7,8^. These results together with other findings suggest that tissue changes in CNP or ET-1 production^9^ may be captured by concurrent changes in plasma if studied under well standardised conditions. Since the CNP gene (*NPPC*) expression is upregulated by statins in at least three different tissues – human umbilical vein endothelial cells^10^, porcine aortic valve interstitial cells^11^ and hepatic endothelial cells cultured from cirrhotic rats^12^, and some of the benefits of statins are lost in the absence of the CNP receptor^11^, further study of interactions of statins with CNP secretion in humans is clearly needed. Endothelin, a pro-inflammatory product of the vascular endothelium, concomitantly inhibited as *NPPC* is upregulated by statins^3^, also deserves closer study especially as the effect of statins on plasma ET-1 is controversial^13–15^.

We have examined possible modulation of CNP peptides and ET-1 by statin therapy, as evidenced by change in their circulating levels in a carefully planned prospective study of i) young and older statin naïve subjects without evidence of renal or cardiovascular disease, and ii) older statin naïve subjects with history of a recent coronary occlusive event. Because CNP production from healthy (atheroma protected) endothelial cells in response to shear stress is much greater than from atheroma prone arterial endothelial cells^16^, we hypothesised that in healthy young adults, statins will acutely increase plasma CNP products whereas responses in statin naive older subjects with overt CAD will differ from age-matched controls without cardiovascular disease. We further posited that the responses of CNP (increase) and ET (decline) to statins would be reciprocal.

## Results

**Figure 1:**
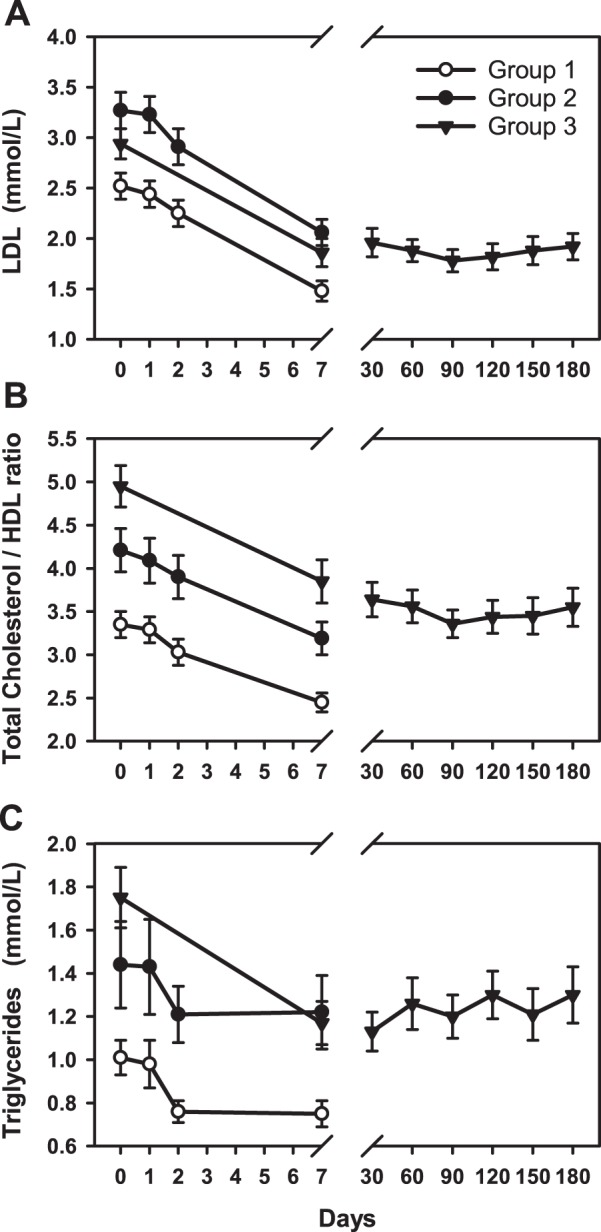
Changes in plasma concentrations of (**A**) low-density lipoproteins, (**B**) total cholesterol/high-density lipoproteins ratio and (**C**) triglycerides in response to 40 mg atorvastatin taken daily commencing Day 0.

**Figure 2:**
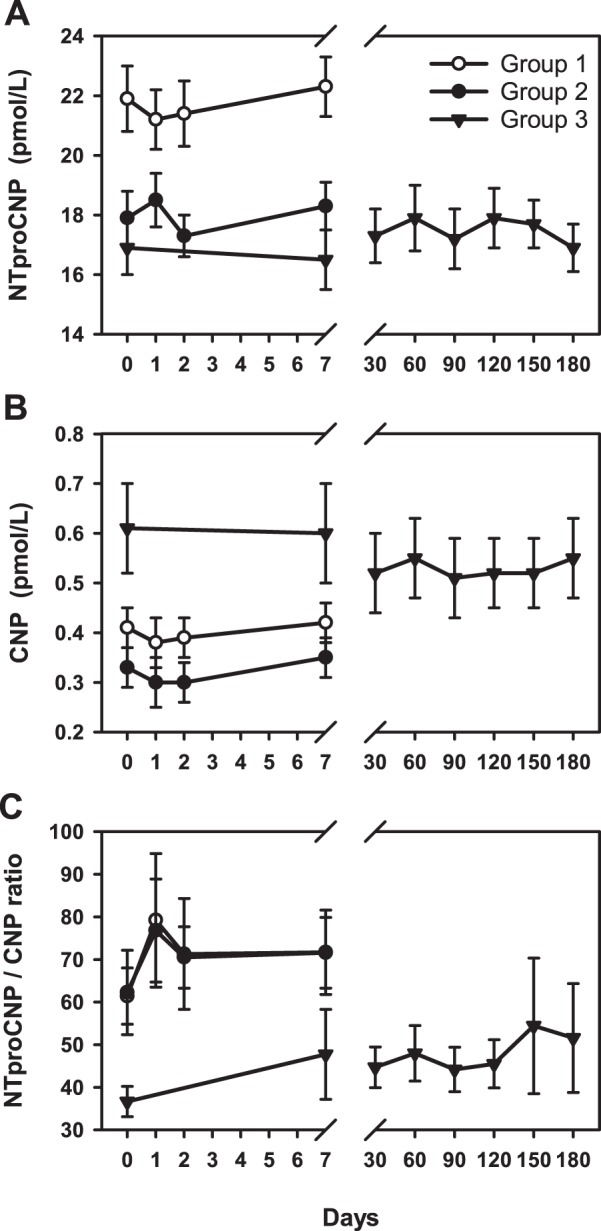
Changes in plasma concentrations of (**A**) NTproCNP, (**B**) CNP and (**C**) NTproCNP/CNP ratio in response to 40 mg atorvastatin taken daily commencing Day 0.

**Figure 3:**
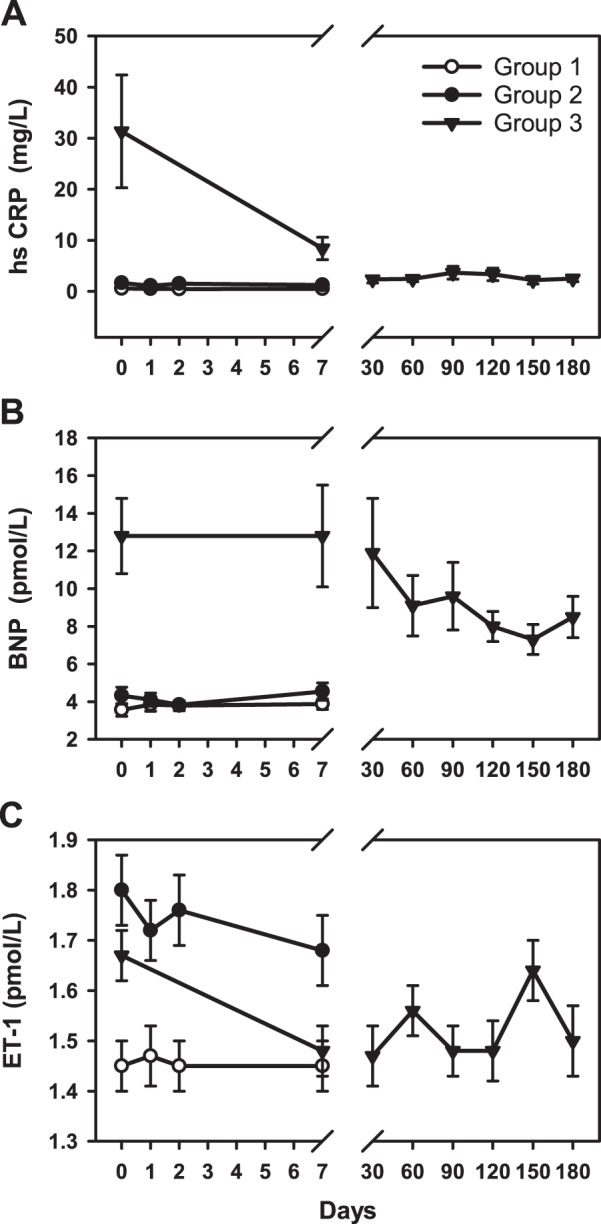
Changes in plasma concentrations of (**A**) hs CRP, (**B**) BNP and (**C**) ET-1 in response to 40 mg atorvastatin taken daily commencing Day 0.

The study was completed without any adverse events. Clinical and biochemical data (medians and inter quartile range) at baseline and day 7 are detailed in Table 1. Compared with older healthy males (Group 2), baseline levels of LDL, Chol/HDL ratio and triglycerides were lower in young males (Group 1). In both groups, all three lipids fell significantly (F < 15, p < 0.001) during statin treatment (Fig. 1). As shown in Fig. 2, mean plasma NTproCNP was higher at baseline in Group 1 compared to Group 2 subjects. Values of both CNP and NTproCNP were stable throughout the study and showed no significant change from baseline values in the week of statin treatment. Adjusting plasma NTproCNP for concurrent serum creatinine did not affect the findings. The ratio of NTproCNP to CNP (NTproCNP/CNP ratio) did not differ between Group 1 and Group 2 subjects and was unaffected by statin treatment (Fig. 2). Plasma BNP at baseline was similar in both groups and was unchanged by statin treatment (Fig. 3). Baseline plasma ET-1 was higher in the older group (F = 9.9, p = 0.003), but in both groups was unchanged by statin treatment (Fig. 3).

**Table 1:** Clinical and biochemical data^†^.

	Group 1			Group 2			Group 3		
	Baseline	Day 7	% change	Baseline	Day 7	% change	Baseline	Day 7	% change
Age (y)	22 (22–24)			52 (49–56)			53 (47–59)		
Body mass index	23 (21–24)			25 (23–30)			31 (28–33)		
Systolic BP (mmHg)	116 (110–122)	114 (110–126)	2%	120 (106–129)	114 (105–137)	0%	130 (120–143)	125 (114–139)	−2%
Diastolic BP (mmHg)	76 (72–80)	72 (70–78)	−4%	79 (71–82)	75 (70–87)	0%	80 (77–89)	79 (74–88)	−1%
Creatinine (umol/L)	94 (88–98)	92 (85–99)	−2%	90 (86–95)	89 (84–95)	0%	89 (81–94)	91 (81–99)	1%
Hs CRP (mg/L)	0.5 (0.2–0.7)	0.4 (0.2–0.7)	−7%	1.3 (0.4–1.8)	1.0 (0.3–1.9)	−16%	9.5 (3.4–20.8)	5.4 (2.5–8.2)*	−42%
Cholesterol (mmol/L)	4.3 (4.0–4.6)	3.1 (2.9–3.2)**	−26%	5.2 (4.7–5.9)	3.7 (3.6–4.2)**	−25%	4.6 (4.1–5.3)	3.3 (2.7–3.9)**	−32%
HDL (mmol/L)	1.3 (1.1–1.4)	1.3 (1.1–1.4)	−1%	1.2 (1.1–1.4)	1.2 (1.1–1.3)	−3%	1.0 (0.9–1.1)	0.9 (0.8–0.9)*	−11%
LDL (mmol/L)	2.5 (2.2–2.7)	1.3 (1.2–1.8)**	−42%	3.5 (2.8–3.9)	2.1 (1.7–2.3)**	−36%	2.9 (2.4–3.4)	1.9 (1.3–2.2)**	−38%
Chol/HDL ratio	3.3 (3.0–3.8)	2.4 (2.2–2.8)**	−27%	4.2 (3.5–4.8)	3.0 (2.6–3.5)**	−23%	5.0 (4.0–5.7)	3.6 (3.5–4.1)**	−25%
Triglycerides (mmol/L)	0.8 (0.7–1.1)	0.7 (0.5–0.9)*	−36%	1.2 (0.7–1.9)	0.8 (0.7–1.5)	−9%	1.6 (1.4–2.1)	1.2 (0.9–1.5)**	−38%
BNP (pmol/L)	2.9 (2.7–3.7)	3.5 (3.0–4.9)	7%	4.2 (3.1–4.8)	4.3 (3.2–5.5)	5%	10 (6–14)	8 (6–12)	0%
CNP (pmol/L)	0.37 (0.30–0.58)	0.44 (0.28–0.53)	−1%	0.34 (0.22–0.42)	0.29 (0.19–0.47)	0%	0.47 (0.36–0.76)	0.48 (0.32–0.65)	−2%
NTproCNP (pmol/L)	20 (15–20)	22 (19–26)	2%	17 (16–20)	18 (16–20)	5%	16 (14–20)	16 (14–18)	−3%
NTproCNP/CNP ratio	52 (40–72)	57 (39–97)	17%	50 (40–71)	64 (44–91)	7%	39 (27–43)	37 (27–53)	0%
ET-1 (pmol/L)	1.4 (1.3–1.6)	1.5 (1.3–1.6)	−3%	1.7 (1.6–2.0)	1.6 (1.5–1.9)	−6%	1.6 (1.5–1.8)	1.5 (1.4–1.6)**	−11%

*p < 0.05, ** p < 0.001 (day 1 versus day 7).

^†^Values are medians and interquartile ranges.

In Group 3 subjects (Table 1), baseline values of HDL were lower (p < 0.001), and the Chol/HDL ratio higher (p = 0.04) than those of the other groups. After 7 days of statin treatment, mean LDL, Chol/HDL ratio and triglycerides were all significantly decreased compared to baseline – and remained so for the study’s duration (Fig. 1). The fall in LDL and the Chol/HDL ratio at day 7 was similar to that of Group 2 subjects. Plasma NTproCNP at baseline in Group 3 was similar to those of age matched healthy controls (Group 2), was unchanged at day 7 and did not change over the 6 months of statin treatment (Fig. 2). These findings were unchanged after adjusting for serum creatinine. However compared to values in Groups 1 and 2, mean plasma CNP in Group 3 subjects was significantly higher (p < 0.01) at baseline, unchanged on day 7 and remained higher than levels in Group 1 or 2 during the 180 day period of statin treatment (Fig. 2). These changes in CNP peptides are reflected in the NTproCNP/CNP ratio which is significantly lower (p < 0.03) at baseline, and subsequently in Group 3 subjects during the 6 month period of statin treatment. Plasma BNP was significantly higher at baseline (p < 0.001) compared to levels in Groups 1 and 2 and declined progressively (p < 0.001) in the 6 month period post ictus. In Group 3 subjects, high sensitivity C Reactive Protein (hsCRP) and BNP were highly correlated (r = 0.6, p = 0.004). Plasma ET-1 concentrations at baseline in Group 3 were not significantly different from levels in Groups 2, fell significantly by day 7 (p = 0.001) but subsequently were unchanged during statin treatment (Fig. 3).

Plasma hsCRP, higher in older than in young healthy subjects (p = 0.02), did not change in either Group 1 or 2 during statin treatment (Fig. 3). Values were raised but highly variable at baseline in Group 3 subjects. Although levels were stabilised – and lower – after one month of statin therapy, hsCRP remained higher than age matched Group 2 subjects throughout the period of study (p = 0.003). Both hsCRP and ET-1 fell within 7 days of statin therapy in Group 3 (p = 0.03 and p = 0.001 respectively), but values at baseline were not correlated (r = 0.23, p = 0.08).

Examining relationships between CNP products and ET-1, in all subjects at baseline (Table 2) an inverse association of ET-1 was found with NTproCNP (r = −0.28, p = 0.03) but the significance of this relationship was lost when the groups were examined individually. Similarly, inverse associations were identified between BNP and NTproCNP, and the ratio NTproCNP/CNP, but not when analysed within each group (Table 2).

**Table 2:** Associations of CNP products with plasma analytes at baseline in all subjects (Groups 1, 2 & 3).

(n = 66)	CNP	NTproCNP	NTproCNP/CNP ratio
BNP	0.19	−0.31*	−0.39**
hs-CRP	0.24	−0.26*	−0.33*
ET-1	−0.03	−0.28*	−0.10
Triglycerides	−0.19	−0.08	0.23
LDL	0.06	−0.13	−0.19
HDL	−0.08	0.12	0.08
Chol/HDL ratio	0.19	−0.18	−0.25

*p < 0.05.

**p < 0.001.

## Discussion

This is the first *in-vivo* study examining the impact of statin therapy on CNP products in any species. In this study circulating levels of CNP products were not significantly modulated by lipid lowering doses of atorvastatin. These findings need to be seen in the context of the many factors contributing to the concentration of plasma CNP products, and the disparate conditions underlying *in vitro* cellular responses from those acting *in vivo*. Compelling evidence from cultured human umbilical vein endothelial cells shows that the transcription factor KLF2, upregulated in response to laminar shear stress^16^ or inflammation^1^, potently stimulates a range of proangiogenic factors including endothelial NO synthase (eNOS) and CNP while inhibiting endothelin-1 expression^3^. In cell culture studies over expressing *KLF2*, CNP was one of the most strongly upregulated genes, and associated with dramatically increased CNP protein levels in the supernatant^3^. Aligning with these findings, and other evidence of CNP’s athero-protective role^17^, athero-prone and athero-protective regions of human carotid arterial wall endothelial cells exhibit differential responses to shear stress: Klf2 expression is more than 5 fold and *Nppc* more than 13 fold enhanced in athero protective regions^16^. Since many of the pleiotropic effects of statins – including anti-inflammatory actions and upregulation of eNOS gene expression^18^ – mimic actions of KLF2 in endothelial cells, formal studies of statin effects have been examined using human umbilical vein endothelial cells^10^. In these studies, dose responsive upregulation of Klf2 by a range of statin drugs was observed after 8 hr and sustained for at least 24 hr. Of the recognised downstream targets of Klf2, upregulation of *Nppc* was not only the most sensitive (increased at 8 hr) but also the most prominent at 24 hr. Inhibiting Klf2 by siRNA nullified the *Nppc* response to statin – suggesting that changes in CNP gene expression induced by statin were Klf2 dependent. Collectively these *in vitro* findings suggest that circulating levels of CNP products may be acutely modulated by statins in humans – and therefore could have applications in drug monitoring as well as providing insight to the integrity of the arterial vasculature^10^. On the basis of the positive link of Klf2/CNP responses in athero protective tissues, compared to the relative lack of response in athero prone tissue, we further posited that the CNP response to statin would be age dependent, and greater in those free of arterial disease compared to those with a recent coronary ischaemic event. Our findings that circulating concentrations of the stable product of proCNP (NTproCNP) – nor the concentration of authentic CNP – did not change during standard (lipid reducing) doses of atorvastatin for periods of 7 days in healthy subjects, and beyond during an extended period of 6 months in those with CAD, lead us to conclude that in males, doses of the drug used in routine clinical practice do not affect plasma CNP products. Exactly the same conclusion can be drawn from the observations that plasma ET-1 is similarly unchanged by statins. Although responses of lipids to atorvastatin are similar in both sexes^19^, whether similar findings apply in statin naïve treated females remain to be studied.

Several factors contributing to the lack of effect of atorvastatin on plasma CNP peptides need consideration. **First**, findings from *in vitro* cell studies (mostly of umbilical venous origin) may not recapitulate physiology^20^. Although *in vivo* studies of the molecular events underpinning vascular homeostasis continue to support the important role of Klf2^2,20^ and/or its downstream targets^21^, with one exception^21^ none have explored the *in vivo* effects of statins on these molecular pathways in arterial tissues from humans^2^. In experimental animals, two studies have specifically addressed the role of CNP in statin’s actions *in vivo*. Using a rat atherosclerosis model of carotid artery intimal thickening^22^, Li *et al*. showed that atorvastatin (10 mg/k/d for 14 days), in reducing intimal thickening, not only increased the arterial tissue content of CNP but also upregulated *Nppc* and its downstream target (cGPK) when compared to vehicle administration. In another setting – hepatic cirrhosis in rats – simvastatin (25 mg/k/d for 3 days) increased *Klf2*, eNOS and *Nppc* gene expression in hepatic sinusoidal endothelial cells^12^. Collectively these results suggest that *in vitro* findings may be reproduced *in vivo* – at least during exceptionally high dose statin administration. **Second**, drug concentration in endothelial tissue and type of statin are likely to be important. Concentrations effective in stimulating *Klf2* and *Nppc* gene expression *in vitro* approximate 100–1000 nmol/L – much higher than mean (24 hr) plasma concentrations (4–33 nmol/L) found during conventional statin therapy^23,24^. However in a pilot study undertaken by us in Sprague Dawley male rats aged 6 weeks (n = 2 for each study), plasma CNP and NTproCNP levels did not differ from vehicle treated controls after receiving simvastatin 10 mg/kg/day by gavage for 24 hr, or 40 mg/kg/day for 7 days. Type of statin also could be important^10^ depending on lipophilicity and membrane permeability. Although a complex issue, evidence suggests that atorvastatin has comparable ability to penetrate cells^25^ when compared to other statins found effective *in vitro*. **Third**, the extent to which changes in endothelial CNP products in tissues affect plasma concentrations is an important issue – particularly in light of the paracrine role of the peptide and the rapid degradation of bioactive CNP at source. Although the bio inactive product NTproCNP is not likely to be degraded at source, whether efflux from extracellular fluids is sufficient to affect plasma concentrations is unclear. While difficult to rigorously assess in humans, our findings from veno-arterial sampling across organs^26,27^, and recent findings linking higher values of plasma NTproCNP (and to a lesser extent, CNP) with vascular stress in adults without history of heart disease^5,6^, support the view that vascular endothelial proCNP does contribute to circulating levels in some settings. Furthermore, in endothelial cell-specific CNP knock out mice, plasma CNP is significantly reduced^9,28,29^. On the basis of these reports we conclude that if concentrations in tissues do indeed increase during statin therapy, any such increase is insufficient to affect plasma levels during the initiation of conventional doses of atorvastatin. Findings relating to plasma ET-1 concentrations are similarly affected by issues 1–3 as discussed above. Although up regulation of endothelin in tissues was reflected by increased plasma ET-1 in mice studies^9^, the degree to which tissue levels impact on plasma ET-1 remains uncertain^30^. Uncertainty is increased by the inconsistent effects of statins on plasma ET-1 levels in humans. A small but significant reduction (mean 0.3 pg/mL corresponding to 0.13 pmol/L) was found in a meta-analysis of 15 controlled clinical trials of subjects – many of whom had serious co morbidities^13^. At least three other studies have found no change in plasma ET-1 levels during statin therapy^14,15,31^. However, none of these studies examined plasma levels within days of commencing therapy. The significant decline at day 7 noted in Group 3 of the current study, and associated fall in hsCRP presumably relates to reduced inflammation post ictus^32,33^. Values after 7 days remained unchanged despite a further fall in hsCRP suggesting that only severe inflammation affects ET-1 expression and circulating levels in this age group. Absence of any change in these endothelial products in plasma during statin therapy, in the face of compelling evidence of their actions *in vitro*, now calls for studies using conventional or higher doses of statin in which tissue protein and gene expression in arteries from experimental animals (or from humans undergoing vascular resections) are made. This is important as current recommended doses of statin, as pointed out by others^1^, have targeted effects on lipids and are not optimised for targeting putative pleiotropic effects such as Klf2 dependent biomarkers which are highly relevant to vascular biology and progression of atherosclerosis^2^. Findings from such studies could be important in selecting type of statin and appropriate dose in clinical practice.

While not the primary objectives, several other findings deserve comment. Higher levels of NTproCNP at baseline in healthy young males than their older counterparts likely reflects the impact of higher skeletal bone turnover in younger subjects, as seen previously^34^. In Group 3 males, compared to other groups, higher levels at baseline of plasma CNP (but not NTproCNP), which are maintained on day 7 and subsequently, is an important finding which resonates with previous results from sequential sampling in subjects with acute coronary syndrome^35^. Similar disproportionate increase in CNP compared to NTproCNP was noted in subjects with heart failure^36^, and in subjects undergoing cardiac catheterisation for acute chest pain^27^. The anomaly has been attributed to competitive interactions among natriuretic peptides in clearance pathways^37,38^ when cardiac secretion of ANP and BNP are increased by cardiac stress. However this explanation seems unlikely in the current study especially in light of the relatively modest elevations in plasma BNP in Group 3 subjects. Provided renal function is normal, NTproCNP concentration will reflect proCNP production in tissues whereas CNP concentrations in plasma – rapidly cleared at source^4^ and from plasma^39^ – are at the limits of detection in healthy adults. Comparing Group 3 levels of CNP products with age matched controls, notably NTproCNP does not differ but CNP is raised in Group 3 post ictus, and the ratio NTproCNP/CNP is much reduced throughout the 6 months of study. In contrast, plasma BNP significantly declines. These findings indicate that post ictus CNP *production* does not change – higher values resulting from reduced CNP *clearance*. The fact that BNP declines whereas CNP does not is consistent with the much lower affinity of human BNP for NPR3^40^ but whether BNP clearance is affected after cardiac injury requires concurrent measurement of both bio and bio-inactive forms. Recent work showing that the clearance receptor NPR3 is down regulated after hypoxic or ischaemic insults in a wide range of cardiac tissues, and remains reduced in established heart failure^41^, provides a plausible mechanism. Alternatively upregulation of osteocrin – a peptide expressed and secreted by the failing human heart^42^ and which has anti-inflammatory actions reducing cardiac remodelling in experimental animals^43^ – could mediate the changes by displacing CNP from NPR3^44^ as shown recently in transgenic mice^45^. Future study of temporal responses of plasma osteocrin in subjects presenting with ACS, and plasma levels of microRNA-100 and miRNA-143 (proven negative regulators of *Npr3* expression in cardiac tissue^41,46^, can be expected to clarify these observations and contribute to our understanding of the adaptive responses to cardiac injury in humans. Although statins are reported to upregulate osteocrin gene expression in bone tissues^47^, any possible contribution of statins to the current findings seems unlikely in view of unchanging NTproCNP/CNP ratios from baseline values in days 1–7 in all three groups.

Our study had several limitations. Only males were studied. While to our knowledge possible effects of sex on Klf2 and downstream targets have not been studied, it cannot be assumed that our findings will apply to females. No placebo/control studies were done, nor was sampling undertaken after cessation of atorvastatin. However the stable levels of both bio and inactive CNP products, the relatively low range of concentrations within each group and unchanging levels in 3 different groups of subjects (66 subjects in toto) militate against a different outcome had these additional interventions been made. No dose-response study in humans was done in light of the lack of effect of very high doses in pilot studies of rats. Conceivably higher dose atorvastatin (80 mg/day) achieving reductions in LDL cholesterol >50% in humans may increase CNP products in plasma. However study of effects of conventional doses used in clinical practice – known to be effective in prevention of cardiovascular disease – was our primary objective. By limiting confounders affecting plasma CNP peptides (such as cardiovascular risk in healthy subjects), variable renal function (correcting for serum creatinine), and variable cardiac status in subjects with overt CAD (measuring plasma BNP and correcting for BNP cross reactivity in the CNP assay), the findings clearly set a limit on the putative actions of statins on CNP production within the healthy and diseased vasculature.

## Materials and Methods

Participants: Lack of females presenting with CAD restricted the study to males. Three groups of male subjects were enrolled for the study. Group 1 (23 subjects) comprised healthy young subjects (age 20–25 yr) without family history (1^st^ and 2^nd^ degree relatives) of coronary artery disease (CAD), stroke or other arterial disease, were non-smokers and had no personal history of high blood pressure, diabetes, renal disease or lipid disorder. None was receiving medication. Body mass index (18–25), systolic (<140 mmHg) and diastolic (<90 mmHg) blood pressure were normal. All had a normal blood lipid profile. Group 2 (21 subjects) were aged 40–60 yr and met the same criteria as detailed for Group 1. Neither group had previously received statin therapy. Group 3 (22 subjects, aged 40–60 yr) were enrolled soon after discharge from hospital once stabilised after an acute coronary event established by coronary angiography. None had previously received statin drugs. Subjects with heart failure, diabetes, metabolic bone disease or recent fracture, or with raised serum creatinine (>110 umol/L) were excluded.

Study procedures: All subjects received atorvastatin 40 mg daily, commencing immediately after overnight fasting blood sampling at 0800 hr. In Groups 1 and 2, the drug was administered daily for 7 days. Venous blood was collected (baseline, day 1, day 2 and day 7) for measurement of CNP products, B-type Natriuretic peptide (BNP), ET-1, lipids (total cholesterol, HDL cholesterol, Chol/HDL ratio, triglycerides), high sensitive C Reactive Peptide (hsCRP) and serum creatinine. In Group 3 participants, atorvastatin 40 mg was administered daily for a period of 6 months. Venous blood was sampled (0800 hr fasting) for the same analytes at baseline, day 7 and then at intervals of one month.

This study was approved by the New Zealand Northern B Health and Disability Ethics Committee (13/NTB/67) and all participants provided written informed consent. All procedures were performed under protocols approved by the New Zealand Northern B Health and Disability Ethics Committee and conducted in accordance with the Declaration of Helsinki. The study is registered with the Australian New Zealand Clinical Trials Registry, number ACTRN12613000525785, registration date 13/5/2013.

Assays: Plasma CNP, NTproCNP and ET-1 following extraction over Sep Pac C18 cartridges were measured by radioimmunoassay as previously described^48,49^. Because there could be significant cross reactivity from BNP in the CNP assay^50^, BNP concentrations were also measured and CNP concentration adjusted accordingly^27^. Only the corrected CNP values are reported here. The molar ratio of NTproCNP to CNP (NTproCNP/CNP) was calculated for each sample by dividing the former by the latter. Intra- and inter-assay CV for CNP and NTproCNP were (4.4% and 8.3% at 1.8 pmol/L) and (6.4% and 8.9% at 18 pmol/L) respectively. Intra- and inter-assay CV for ET-1 and BNP were (6.1% and 9.6% at 1.3 pmol/L) and (5.4% and 8.3% at 9.7 pmol/L) respectively. Standard methods were used to measure plasma lipids, serum creatinine and CRP (Abbott Architect c16000, Abbott Diagnostics, Illinois, U.S.A.).

Statistical analyses: Data are presented as mean ± sem unless otherwise stated. Repeated measures ANOVA was used to assess changes in plasma markers among groups and across time. Where significant changes were observed with ANOVA, Bonferroni post hoc analysis was used to detect differences from baseline time 0 values and inter group time-matched data as appropriate. BNP and hsCRP data were log10-transformed to satisfy parametric assumptions. Spearman correlation coefficients were used to test the associations between plasma CNP products with other indices. All tests were two sided and statistical significance was assumed when P < 0.05.

## Data Availability

The datasets analysed during the current study are available from the corresponding author on reasonable request.
